# Regulatory Mechanism through Which Old Soybean Leaves Respond to Mn Toxicity Stress

**DOI:** 10.3390/ijms25105341

**Published:** 2024-05-14

**Authors:** Yuhu Pan, Jianning Shi, Jianyu Li, Rui Zhang, Yingbin Xue, Ying Liu

**Affiliations:** 1 Department of Biotechnology, College of Coastal Agricultural Sciences, Guangdong Ocean University, Zhanjiang 524088, China; 2 Department of Agronomy, College of Coastal Agricultural Sciences, Guangdong Ocean University, Zhanjiang 524088, China

**Keywords:** *Glycine max*, old leaves, manganese poisoning, molecule mechanism, hormone regulation

## Abstract

Manganese (Mn) is a heavy metal that can cause excessive Mn poisoning in plants, disrupting microstructural homeostasis and impairing growth and development. However, the specific response mechanisms of leaves to Mn poisoning have not been fully elucidated. This study revealed that Mn poisoning of soybean plants resulted in yellowing of old leaves. Physiological assessments of these old leaves revealed significant increases in the antioxidant enzymes activities (peroxidase (POD), superoxide dismutase (SOD), ascorbate peroxidase (APX), and catalase (CAT)) and elevated levels of malondialdehyde (MDA), proline, indoleacetic acid (IAA), and salicylic acid (SA), under 100 μM Mn toxicity. Conversely, the levels of abscisic acid (ABA), gibberellin 3 (GA_3_), and jasmonic acid (JA) significantly decreased. The Mn content in the affected leaves significantly increased, while the levels of Ca, Na, K, and Cu decreased. Transcriptome analysis revealed 2258 differentially expressed genes in the Mn-stressed leaves, 744 of which were upregulated and 1514 were downregulated; these genes included genes associated with ion transporters, hormone synthesis, and various enzymes. Quantitative RT-PCR (qRT-PCR) verification of fifteen genes confirmed altered gene expression in the Mn-stressed leaves. These findings suggest a complex gene regulatory mechanism under Mn toxicity and stress, providing a foundation for further exploration of Mn tolerance-related gene regulatory mechanisms in soybean leaves. Using the methods described above, this study will investigate the molecular mechanism of old soybean leaves’ response to Mn poisoning, identify key genes that play regulatory roles in Mn toxicity stress, and lay the groundwork for cultivating high-quality soybean varieties with Mn toxicity tolerance traits.

## 1. Introduction

The sources of manganese (Mn) in the soil include emissions from industrial production, agricultural activities, and mineral mining [1,2]. As a result of recurrent human interventions, Mn increasingly infiltrates groundwater or accumulates at the soil surface [3]. Within the soil matrix, Mn exists in three oxidation states: soluble Mn (Mn^2+^), which is absorbable by plants, and insoluble Mn (Mn^3+^ and Mn^4+^), which remains inaccessible to plants [4]. Statistical data indicate an annual release of approximately 1500 tons of Mn into the environment, with a substantial portion existing as soluble Mn (Mn^2+^) in the soil [5]. Furthermore, soil acidification elevates the Mn content, with levels ranging from 20 to 3000 milligrams per kilogram of soil and a mean concentration of probably 600 milligrams per kilogram [6]. Excessive Mn accumulation poses environmental contamination risks and hampers plant growth [7,8]. For instance, corn (*Zea mays*) and sunflower (*Helianthus annuus*) encounter Mn toxicity and growth impediments when Mn accumulates above 200 μg/g (dry weight) and 5300 μg/g (dry weight), respectively [9].

Mn-induced plant poisoning manifests as various symptoms, including diminished root branching and the formation of Mn deposits on stems and leaves [10,11]. Predominantly, Mn toxicity disrupts the thylakoid structure within chloroplasts, diminishing energy capture by leaves and its transfer to the photoreaction center, thereby compromising photosynthetic electron transport efficiency and inciting photoinhibition [12,13]. Photoinhibition triggers oxidative stress in plants, leading to the generation of an abundance of ROS (reactive oxygen species), containing^1^O_2_ (singlet oxygen), H_2_O_2_ (hydrogen peroxide), and O_2_^−^ (superoxide radicals) [14,15]. These oxides initiate the degradation of membrane lipids, proteins, carbohydrates, and nucleic acids; disrupt cellular osmotic equilibrium; and ultimately culminate in plant death [15,16]. Additionally, excessive Mn can interfere with the assimilation and transport of other vital ions, such as P (phosphorus), Fe (iron), Mg (magnesium), and Ca (calcium) [17]. Furthermore, prolonged consumption of Mn-rich foods by humans may result in neurodegenerative diseases and impact the proper functioning of various physiological systems [18,19].

Plants have evolved diverse physiological and molecular mechanisms to counteract Mn toxicity. These mechanisms include ion uptake within the rhizosphere, intracellular Mn transport, activation of antioxidant enzymes, and increased secretion of acidic organic compounds to mitigate the harmful effects of available Mn [20]. For instance, Mn toxicity stress can induce extraneous Mn sequestration within cowpea plants (*Vigna sinensis*), concurrent with an increase in in vivo antioxidant enzyme activity and excess Mn oxidation [21,22]. These findings imply that Mn transport to vacuoles may constitute a crucial mechanism for enhancing Mn tolerance in plant leaves [23]. Numerous transporters, including *Oryza sativa* OsMTP8.1, *Cucumis sativus* CsMTP8, *Stylosanthes guianensis* SgMTP8, and *Arabidopsis thaliana* AtMTP11, have been found to be involved in isolating Mn from vacuoles and participating in Mn detoxification. These discoveries suggest that Mn transporters may exert regulatory control over plant Mn tolerance [23,24,25,26]. Furthermore, plants can bolster their Mn tolerance by secreting acidic organic compounds capable of chelating Mn [27,28]. For instance, the secretion of oxalates, carboxylates, and citrate in perennial ryegrass (*Lolium perenne*) limits Mn uptake and enhances Mn toxicity tolerance [27]. Additionally, root systems enhance plant resistance to Mn toxicity through the modulation of the absorption of mineral elements, containing zinc (Zn), lead (Pb), Fe, Ca, and Mg [17,29].

The soybean plant (*Glycine max*) is a pivotal staple crop in China, where its growth and development are notably susceptible to metal-induced stress. This susceptibility not only curtails soybean yield but also poses a potential risk to consumer health [30,31]. The threshold for Mn (Mn^2+^) toxicity in soybean seedling growth was identified at 0.05 mM. Surpassing this threshold hampers soybean development, primarily by affecting ion absorption and translocation, inciting the generation of ROS, and disrupting hormone homeostasis [32,33,34]. Currently, the specific mechanism governing soybean plants’ response to Mn toxicity stress has not been fully elucidated, particularly with regard to the response of old leaves when suffered from Mn poisoning, a subject that has yet to be explored in the literature. Within plant structure, leaves are universally recognized as the most critical site for photosynthesis [35]. Studies have indicated that the prevalence of Mn oxide spots on old soybean leaves surpasses that on the leaves of all other ternate compound leaves suffered from Mn poisoning [32]. The underlying physiological and molecular responses responsible for this phenomenon have not been fully elucidated. Transcriptome sequencing provides an effective avenue for further dissecting plant response mechanisms under stress conditions. It has been extensively employed in various plant species, including investigations into the response mechanisms of *Cucumis sativus* and *Citrus reticulata* to metal toxicity, such as copper (Cu) and Ca [36,37]. Consequently, in this study, high-throughput screening was employed to implement an overall analysis of the whole-genome transcriptome and comparative assessment of DEGs (differentially expressed genes) in old leaves of soybean plants responded to Mn poisoning. Concurrently, we determined key physiological parameters, ion levels, and hormone concentrations under varying Mn concentrations. These analyses shed light on the specific signaling pathways regulating Mn toxicity in old leaves. Ultimately, by revealing the molecular underpinnings of the response to Mn toxicity in old leaves, we identified pivotal genes implicated in Mn tolerance. Through the above research methods, this study will explore the molecular mechanism of old soybean leaves response to Mn poisoning, further discover some key genes playing regulatory roles in Mn toxicity stress, and lay a foundation for cultivating high-quality soybean varieties with Mn toxicity tolerance traits.

## 2. Results

### 2.1. Effect of Mn Toxicity Stress on Pigment Content of Old Soybean Leaves

**Figure 1:**
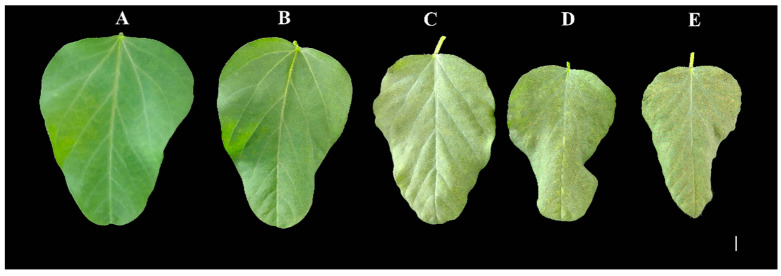
Phenotype of old soybean leaves. The phenotype of old soybean leaves after 15 days treated with various concentrations of Mn (**A**–**E**: 5, 35, 100, 165, 230 µM), Bar = 0.6 cm.

**Figure 2:**
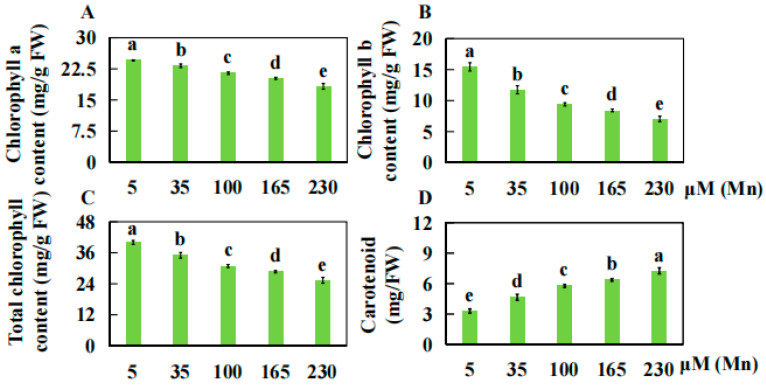
Results of various concentrations of Mn on the pigment content of old leaves. Contents of chlorophyll a (**A**) and b (**B**), (**C**) total chlorophyll, (**D**) carotenoid. Experimental data are displayed as mean value ± SD (standard deviation) from four biological repetitions. Duncan multiple comparison was adopted to analyze the significance of data differences. The columnar chart marked with different lowercase letters demonstrates significant differences between data (*p* < 0.05).

To study the effect of Mn poisoning on pigment composition of old soybean leaves, an analysis of the contents of chlorophyll a and b, as well as carotenoid, was conducted across the various Mn treatment concentrations (Figure 1A–E and Figure 2A–D). Conversely, the carotenoid content displayed the opposite trend. Specifically, in comparison to those in plants treated with a standard Mn concentration of 5 µM, the chlorophyll a content in old leaves was reduced by 5.31%, 12.66%, 17.67%, and 25.55% when plants were subjected to elevated Mn concentrations (35, 100, 165, and 230 µM) (Figure 2A). Similarly, chlorophyll b decreased by 23.95%, 39.14%, 45.49%, and 54.34%, respectively (Figure 2B), while the overall chlorophyll content decreased by 12.50%, 22.88%, 28.41%, and 36.66%, respectively (Figure 2C). In stark contrast, carotenoid levels increased by 40.95%, 74.95%, 93.04%, and 120.08%, respectively (Figure 2D).

### 2.2. Effects of Different Concentrations of Mn on the Physiological Response Indicators of Old Soybean Leaves

The investigation of the influence of Mn poisoning on the various physiological indices of old leaves of soybean plants included measurements of activities of four antioxidases (peroxidase (POD), superoxide dismutase (SOD), ascorbate peroxidase (APX), and catalase (CAT)), soluble protein and sugar levels, proline content, and malondialdehyde (MDA) concentrations in old leaves exposed to varying Mn concentrations.

**Figure 3:**
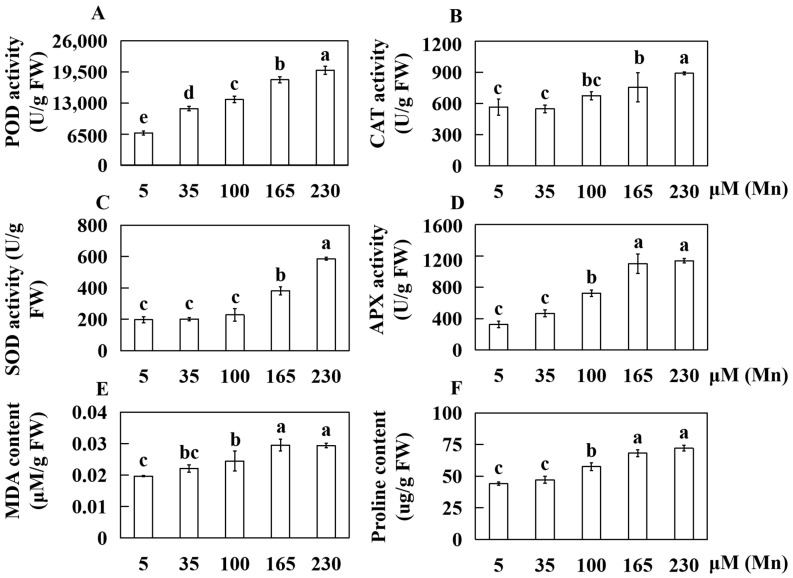
Determination results of physiological response indexes of old soybean leaves treated with different Mn concentrations. Activities of (**A**) POD (peroxidase), (**B**) CAT (catalase), (**C**) SOD (superoxide dismutase), (**D**) APX (ascorbate peroxidase); contents of (**E**) MDA (malondialdehyde), (**F**) proline. Experimental data are displayed as mean value ± SD (standard deviation) from four biological repetitions. Duncan multiple comparison was adopted to analyze the significance of data differences. The columnar chart marked with different lowercase letters demonstrates significant differences between data (*p* < 0.05).

Specifically, when contrasted with the standard Mn concentration of 5 µM, the POD activity in old leaves exhibited marked increases of 75.31%, 103.70%, 164.81%, and 194.14% under elevated Mn concentrations (35, 100, 165, and 230 µM) (Figure 3A). Similarly, SOD activity increased by 2.19%, 15.62%, 93.44%, and 196.88%, respectively (Figure 3C), while APX activity increased by 43.59%, 123.08%, 238.46%, and 250.26%, respectively (Figure 3D). Catalase (CAT) activity increased by 19.12%, 33.82%, and 57.35% at Mn concentrations of 100, 165, and 230 µM, respectively (Figure 3B). Furthermore, compared to that in the standard Mn treatment (5 µM Mn), the MDA content increased by 12.34%, 24.17%, 50.12%, and 49.32% at Mn concentrations of 35, 100, 165, and 230 µM, respectively (Figure 3E). Similarly, the proline content increased by 7.08%, 30.66%, 54.83%, and 63.68%, respectively (Figure 3F).

### 2.3. Effect of Mn Toxicity Stress on the Hormone Content of Old Soybean Leaves

**Figure 4:**
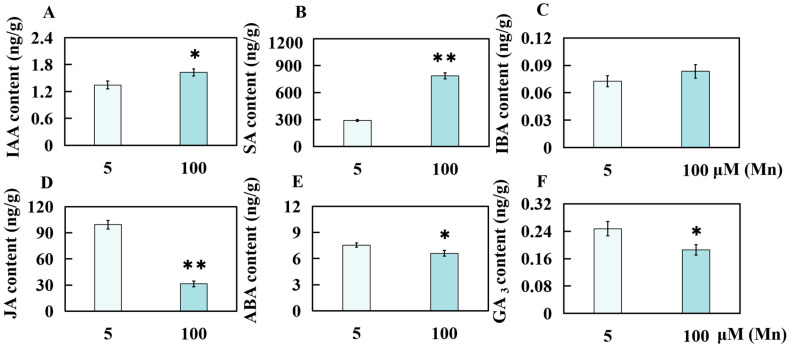
Results of Mn poisoning on the hormone content of old leaves in soybean. Contents of (**A**) indoleacetic acid (IAA), (**B**) salicylic acid (SA), (**C**) indolebutyric acid (IBA), (**D**) jasmonic acid (JA), (**E**) abscisic acid (ABA), (**F**) gibberellin 3 (GA_3_). Experimental data are displayed as mean value ± SD (standard deviation) from three biological repetitions. *t*-test was adopted to analyze the significance of data differences. The columnar chart marked with an asterisk (*) demonstrates significant differences between data (* *p* < 0.05, ** *p* < 0.01).

The examination of old soybean leaves under two Mn concentrations, 5 and 100 µM Mn, facilitated the determination of the contents of key hormones, including ABA (abscisic acid), SA (salicylic acid), IAA (indoleacetic acid), JA (jasmonic acid), IBA (indole butyric acid), and GA_3_ (gibberellin 3). In comparison to those subjected to the standard Mn concentration of 5 µM, the old leaves subjected to an elevated Mn concentration (100 µM) exhibited notable changes in hormone content. Specifically, the IAA, SA, and IBA contents in the old leaves of soybean plants increased by 20.88%, 170.25%, and 14.66%, respectively, under high Mn concentration stress (Figure 4A–C). Conversely, the levels of JA, ABA, and GA_3_ decreased by 68.46%, 12.63%, and 24.83%, respectively, under the same conditions (Figure 4D–E).

### 2.4. Influences of Mn Poisoning on the Ion Contents of Old Leaves in Soybean

**Figure 5:**
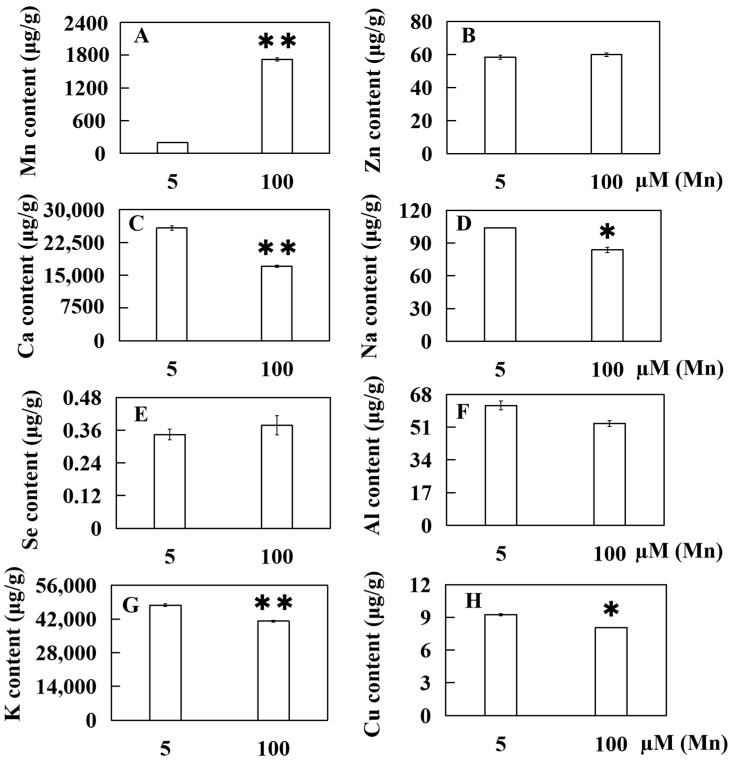
Effect of ion accumulation in old soybean leaves under Mn toxicity stress. The ion contents of (**A**) manganese (Mn), (**B**) zinc (Zn), (**C**) calcium (Ca), (**D**) sodium (Na), (**E**) selenium (Se), (**F**) aluminum (Al), (**G**) kalium (K), and (**H**) cuprum (Cu) were measured. Experimental data are displayed as mean value ± SD (standard deviation) from three biological repetitions. *t*-test was adopted to analyze the significance of data differences. The columnar chart marked with an asterisk (*) demonstrates significant differences between data (* *p* < 0.05, ** *p* < 0.01).

Following the transplantation of soybean seedlings into nutrient solutions with either a standard Mn concentration (5 µM) or a heightened Mn concentration (100 µM) treatment for fifteen days, the Mn, Ca, Se, Zn, K, Na, Cu, and Al contents in old leaves were evaluated. Taken together, these findings revealed that Mn toxicity indeed exerted a discernible effect on the ion content in old leaves of soybean plants (Figure 5). In comparison to that in a standard Mn concentration (5 μM), the Mn content in the high Mn treatment group substantially increased by 769.00% (100 μM) (Figure 5A). Moreover, the Zn and Se contents exhibited slight increases of 2.62% and 9.79%, respectively, although these changes were not statistically significant (Figure 5B,E). Conversely, the contents of Ca, Na, K, and Cu significantly decreased by 33.84%, 19.27%, 13.95%, and 12.82%, respectively (Figure 5C,D,G,H). Although the Al content decreased by 15.03%, the data had not yet reached the level of significant differences (Figure 5F).

### 2.5. Transcriptome Sequencing Analyzing of Old Leaves in Soybean Dealt with Normal or High Mn

**Figure 6:**
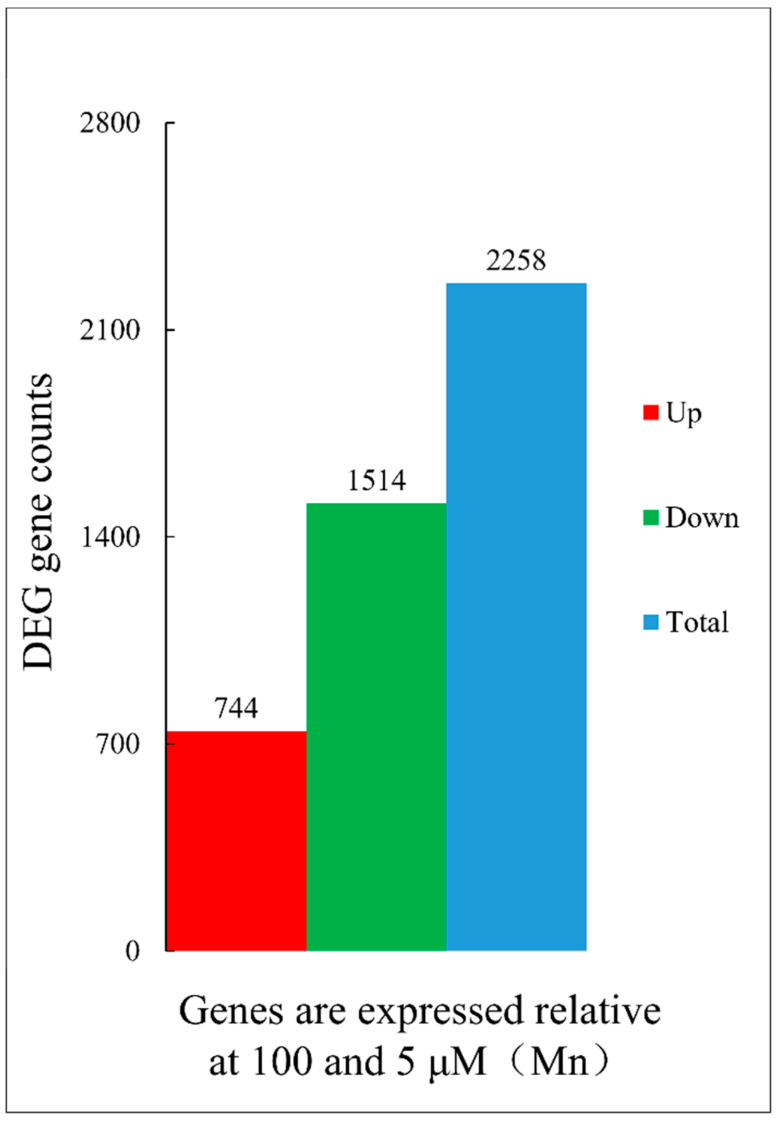
Statistical histogram of DEGs. The red bar chart displays the number of upregulated DEGs (744) (log2 ≥ 1), the green bar chart displays the number of downregulated DEGs (1514) (log2 ≤ −1), and blue shows the total amount of DEGs (2258).

A comprehensive approach involving a genome-wide RNA sequencing technique was adopted to scrutinize the transcriptional activity of old leaves of soybean plants subjected to Mn treatment, specifically at concentrations of 5 μM and 100 μM, over a 15 d period. This research sought to unravel the complex gene regulatory systems orchestrating the response of old leaves of soybean plants to Mn-induced stress. The results of this investigation revealed the presence of a total of 2258 DEGs in old leaves of soybean plants exposed to Mn toxicity stress (Table S1). Within this cohort of DEGs, 744 were upregulated, while 1514 were downregulated (Figure 6).

### 2.6. Identification of Hormone-Related Genes

In comparison to those in the normal Mn treatment, a total of 35 DEGs linked to hormone synthesis were identified within the old leaves of soybean plants under Mn toxicity stress (Table 1). Among these DEGs, 9 genes associated with auxin synthesis were upregulated, specifically, *Glyma.01G137500*, *Glyma.03G029600*, *Glyma.06G158700*, *Glyma.07G043000*, *Glyma.07G051700*, *Glyma.10G151000*, *Glyma.12G035800*, *Glyma.15G076500*, and *Glyma.10G180000*. Conversely, 12 auxin-related genes were downregulated, which included *Glyma.02G125600*, *Glyma.12G071000*, *Glyma.13G150100*, *Glyma.17G046200*, *Glyma.17G165300*, *Glyma.02G007300*, *Glyma.13G354100*, *Glyma.15G012700*, *Glyma.02G142500*, *Glyma.03G158700*, *Glyma.04G046900*, and *Glyma.19G161000*. Furthermore, 11 genes related to gibberellin synthesis were downregulated (*Glyma.06G185300*, *Glyma.09G095200*, *Glyma.14G087200*, *Glyma.17G258200*, *Glyma.02G297700*, *Glyma.06G265500*, *Glyma.08G325900*, *Glyma.12G137700*, *Glyma.12G216100*, *Glyma.17G007600*, and *Glyma.18G081100*), while only three genes were upregulated (*Glyma.12G018100*, *Glyma.15G037200*, and *Glyma.17G160800*).

### 2.7. Identification of Glutathione Metabolism Genes

Fifteen DEGs associated with the metabolism of glutathione were identified within old leaves of soybean seedlings suffered from both normal and heightened Mn treatments (Table 2). Among these DEGs, a total of 4 *glutathione S-transferases* were upregulated, including *Glyma.02G024600*, *Glyma.02G024800*, *Glyma.06G193500*, and *Glyma.15G251900*. Additionally, there were 9 downregulated *glutathione S-transferases*, specifically *Glyma.01G106100*, *Glyma.04G107500*, *Glyma.07G139700*, *Glyma.07G139800*, *Glyma.07G140400*, *Glyma.15G251500*, *Glyma.15G251600*, *Glyma.15G251700*, and *Glyma.18G190200*. Furthermore, two *Glutathione peroxidases*, *Glyma.05G207100* and Glyma.11G024000, were also downregulated in response to Mn treatment.

### 2.8. Identification of Cellulose Biosynthesis Genes

Among the DEGs identified in the old leaves of soybean plants, 16 were associated with cellulose synthase, 4 of which were upregulated, while the remaining 12 were downregulated (Table 3). The upregulated DEGs included four *cellulose synthases*, specifically *Glyma.06G324300*, *Glyma.06G307900*, *Glyma.13G310300*, and *Glyma.04G255400*. Conversely, the downregulated DEGs consisted of 12 *cellulose synthases* (*Glyma.04G063800*, *Glyma.06G065000*, *Glyma.06G225400*, *Glyma.08G088400*, *Glyma.08G117500*, *Glyma.09G051100*, *Glyma.12G191700*, *Glyma.15G157100*, *Glyma.01G232500*, *Glyma.11G010400*, *Glyma.06G225500*, and *Glyma.02G205800*).

### 2.9. Identification of Genes Related to Amino Acid Transport

A comprehensive set of 9 genes related to amino acid transport was identified in the old leaves of soybean plants (Table 4). Among these genes, 2 exhibited upregulation (*Glyma.05G043100* and *Glyma.14G010300*). Conversely, there were 7 downregulated DEGs, including *Glyma.01G084800*, *Glyma.02G260100*, *Glyma.09G238100*, *Glyma.10G201600*, *Glyma.11G097000*, *Glyma.11G226000*, and *Glyma.20G188800*.

### 2.10. Identification of Glycometabolism Genes

Eleven DEGs associated with glycometabolism were identified within old leaves of soybean seedlings suffered from both normal and heightened Mn treatments (Table 5). Among these DEGs, all of the 11 *glycometabolisms* were downregulated, including *Glyma.02G075000*, *Glyma.07G086000*, *Glyma.07G189500*, *Glyma.08G009900*, *Glyma.11G004800*, *Glyma.15G071800*, *Glyma.16G138800*, *Glyma.16G156800*, *Glyma.08G135800*, *Glyma.11G119500*, and *Glyma.13G284900*.

### 2.11. qRT-PCR Verification

**Figure 7:**
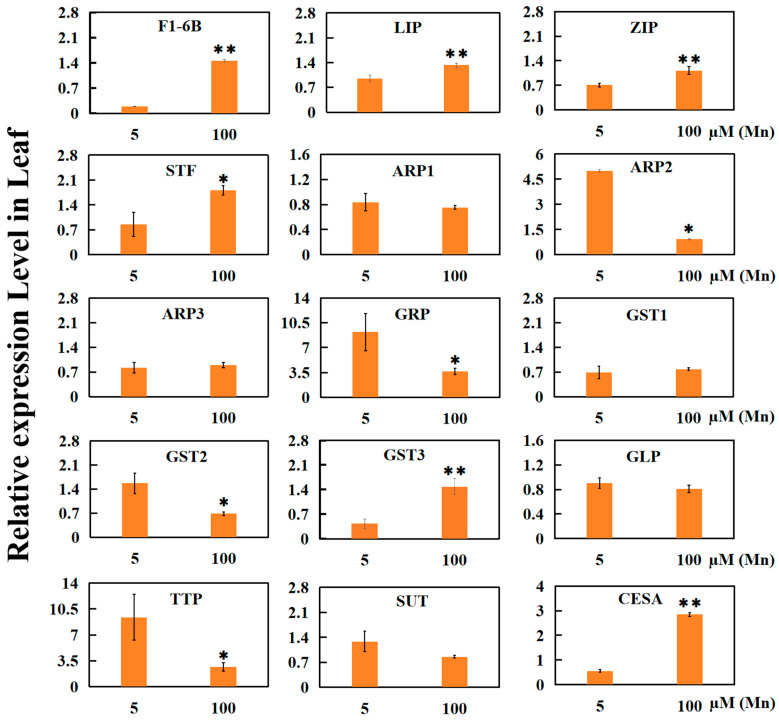
Results of qRT-PCR of 15 genes in old leaves of soybean treated with normal group (5 µM Mn) and treatment group (100 µM Mn). Experimental data are displayed as mean value ± SD (standard deviation) from three biological repetitions. T-test was adopted to analyze the significance of data differences. The columnar chart marked with an asterisk (*) demonstrates significant differences between data (* *p* < 0.05, ** *p* < 0.01). F1-6B: fructose-1-6-diphosphatase; LIP: lipoxygenase; ZIP: zinc transporter; STF: sulfate transporter; ARP: auxin-reactive protein; GRP: gibberellin regulatory protein; GST: glutathione S-transferase; GLP: glutathione peroxidase; SUT: sugar transporter; TTP: transmembrane amino acid transporter; CESA: cellulose synthase.

In the verification of 15 DEGs from old soybean leaves treated with 5 µM or 100 µM Mn through qRT-PCR analysis (Figure 7), several key findings were observed. Among the evaluated gene transcripts, 10 exhibited significant upregulation or downregulation in response to Mn stress, while 5 gene exhibited nonsignificant upregulation or downregulation. Notably, two genes associated with enzymes, including fructose-1-6-diphosphatase (F1-6B) and lipoxygenase (LIP), exhibited significant upregulation. Additionally, two genes related to ion transport were significantly upregulated, namely zinc transporter (ZIP) and sulfate transporter (STF).

Furthermore, two hormone-related genes, namely, auxin reactive protein (ARP2) and gibberellin regulatory protein (GRP), were significantly downregulated. Additionally, two glutathione S-transferase genes exhibited significantly differential expression: the GST2 downregulated, while the GST3 upregulated. The expression of one gene related to sugar transporter (SUT) was not significantly downregulated. In addition, one gene associated with transmembrane amino acid transporter (TTP) exhibited significant downregulation, while one gene associated with cellulose synthase (CESA) exhibited significant upregulation. These findings provide support for the results obtained from RNA-seq (transcriptome sequencing).

## 3. Discussion

The excessive accumulation of available Mn has emerged as a limiting factor for crop growth and development in acidic soils [38]. It is important to note that tolerance to Mn stress varies significantly among different plant species and varieties due to differences in plant structure and nutrient uptake mechanisms. For instance, peanut plants (*Arachis hypogaea*) exhibit lower susceptibility to Mn toxicity than do soybean plants [38,39]. All parts of plants, including roots, stems, and leaves, play vital roles in growth and development. However, the specific mechanisms governing the responding of old plant leaves to Mn toxicity have not been clarified. Previous research reports have also revealed that high Mn stress restrains the development of plants such as peanuts (*Arachis hypogaea*) and golden rain trees (*Koelreuteria paniculate*), resulting in a significant decrease in leaf biomass [38,40]. In this study, Mn treatment hindered the development of old soybean leaves, leading to a reduction in biomass and pigment content. The decrease in photosynthetic pigment content may be attributed to Mn binding with protein components in soybean leaf chloroplasts, while the increase in ROS under Mn stress could damage chloroplast structure and function [41]. Furthermore, Mn accumulation might compromise the integrity of plant cells, disrupt the synthesis of photosynthetic pigments, hinder plant photosynthesis [40], and ultimately affect the development of old leaves in soybean.

In response to abiotic stress, plants generate a significant amount of ROS, which can profoundly affect normal plant growth and development. Therefore, it is crucial to regulate the balance between ROS production and removal in plants [7,38]. Antioxidant enzymes play a pivotal role in this regulation by participating in the detoxification of ROS and maintaining this balance. The key enzymes involved in this process include CAT (catalase), SOD (superoxide dismutase), APX (ascorbate peroxidase), and POD (peroxidase) [7,39]. POD is a common and highly active enzyme in plants, which catalyzes the REDOX reaction using H_2_O_2_ as an oxidant, and reduces H_2_O_2_ to HO_2_ while oxidizing other substances, which is used to remove H_2_O_2_ in cells, and is one of the protective enzymes in plants [7,39]. Increasing the activity of antioxidant enzymes can enhance a plant’s ability to detoxify ROS, reducing oxidative damage caused by Mn toxicity stress [42].

The results of the current study indicate that the activities of APX, POD, CAT, and SOD increased significantly with increasing Mn concentration. These findings suggested that the antioxidant enzyme defense system in old soybean leaves is rapidly activated responding to Mn poisoning. This mechanism aligns with the stress mitigation mechanisms observed in other crops, including peanuts, under heavy metal stress [43].

MDA is a product of membrane lipid peroxidation and serves as a vital index of the extent of LPO (lipid peroxidation) in plant cytomembrane [44]. Elevated MDA levels can transform amino acids, proline, and nucleic acids into insoluble compounds, disrupting normal tissue cell function [44]. Previous studies have demonstrated that plant tissues can function properly under moderate heavy metal stress [45]. However, once the stress intensity surpasses a certain threshold, it can lead to an increase in ROS levels, metabolic disruptions, membrane lipid peroxidation, and an increase in the MDA content in tissue cells [45]. In the current study, the content of MDA in old soybean leaves increased significantly with increasing Mn concentration. These findings suggested that Mn stress may trigger the production of a substantial amount of ROS in old leaves, leading to cell membrane system damage and a significant increase in MDA content.

Proline has essential functions as a plant osmotic regulator and a scavenger of reactive oxygen free radicals, helping to maintain a dynamic balance between tissue cell osmotic regulation, free radical production, and detoxification [46,47]. In this study, an obvious increasing in the proline content in old soybean leaves was observed with increasing Mn concentration. Taken together, these findings indicate that old soybean leaves require relatively high proline levels to regulate the tissue cell osmotic balance and actively detoxify oxygen free radicals under Mn toxicity stress.

Plant hormones are chemical messengers crucial for regulating plant stress responses and growth and development [48]. In the context of heavy metal stress, plant hormones enhance plant resistance to heavy metal toxicity by activating various molecules involved in regulatory and signaling pathways [39]. Within this framework, the *PINFORMED* and *AUXIN1/LIKE AUX1* play roles in regulating the transport of growth hormones, which are vital for the growth and development of plant tissues [38]. Mn toxicity can reduce the root auxin concentration and inhibit root growth by downregulating the expression of the auxin effector genes *PIN4* and *PIN7* [11]. In the present study, old soybean leaves exhibited a significant increase in auxin concentration after exposure to Mn toxicity, which differs somewhat from previous findings. This variation may be attributed to evolutionary differences between species and the influence of different treatments.

Gibberellic acid (GA) is a tetracyclic diterpenoid compound that regulates various aspects of plant tissue growth and development, including stem elongation, leaf expansion, and sprout germination, as well as responses to environmental stress [49]. The GA gene family participates in the biosynthesis and degradation of GA_3_, influencing leaf aging rates [49]. Upregulation of the GA_2_ oxidase gene, which inhibits GA activity during leaf senescence, was observed, indicating that a reduction in GA may contribute to leaf senescence [49]. Additionally, GA_3_ has been shown to reduce the expression of *IRT1* (a gene related to Cd absorption) and mitigate toxic symptoms caused by cadmium poisoning in Arabidopsis [50]. Furthermore, GA_3_ enhances salt stress resistance by increasing the SA concentration in *Arabidopsis thaliana* [51]. However, prior studies have also indicated that the endogenous GA content decreases with leaf aging [49]. In this study, the content of GA_3_ in old soybean leaves significantly reduced after Mn toxicity, possibly due to the accelerated aging of old leaves induced by Mn toxicity, which led to the inhibition of GA_3_ synthesis.

SA is a pivotal phenol-related hormone involved in plant metabolism and responses to environmental stress [49]. The SA content tends to increase as plant leaves age [52]. Studies have demonstrated that SA biosynthesis is inhibited in functionally deficient Arabidopsis SA mutants (*sid2*), leading to a delay in leaf senescence [53]. Therefore, maintaining SA homeostasis is critical for promoting leaf aging, and various WRKY transcription factors have been shown to modulate the SA signaling pathway in different ways to regulate leaf aging [49]. For instance, *WRKY75*, *WRKY46*, and *WRKY63* enhance the expression of *SID2* (a SA biosynthesis gene) by binding to its promoter, thereby increasing leaf SA content and promoting the leaf aging process [49]. Additionally, JA is derived from polyunsaturated fatty acids and is an oxophospholipid plant hormone that mediates components of the signaling pathway to regulate leaf senescence [49]. There is a complex relationship between JA and SA in the regulation of leaf senescence [54]. For instance, *WRKY53* positively regulates leaf aging, but its function is inhibited by JA-induced ESR/ESP, both of which regulate SA and JA homeostasis to mediate the leaf aging process [54]. Moreover, low concentrations of SA upregulate autophagy, which in turn slows JA-induced leaf senescence [55]. Hence, an antagonistic relationship between SA and JA during leaf senescence has been established [55]. In the current study, the content of SA in old soybean leaves significantly increased after Mn exposure, while the JA content decreased significantly. These findings indicate that the antagonistic relationship between SA and JA in old soybean leaves might be one of the mechanisms for alleviating Mn poisoning and maintaining the internal environmental homeostasis of old soybean leaves. However, it is worth noting that the relationships between SA and JA, as well as their interactions with other hormones, are complex and require further in-depth study and discussion.

Transcriptome sequencing has become a widely adopted approach for investigating plant responses to heavy metal stress [38,56]. However, the response of peanut and rice leaves to Mn stress has only recently been reported [38,56]. Transcriptome results have indicated the presence of 4589 and 2831 genes in peanut [38] and Mn-sensitive rice [56] leaves, respectively, suggesting that those two crops perhaps have various response mechanisms to cope with Mn poisoning stress. Nevertheless, there have been limited reports on soybean leaves in this context. In the current study, transcriptome sequencing technology was adopted to analyze the entire genomics of old soybean leaves under Mn stress conditions. All 2258 DEGs were discovered in the old soybean leaves, 744 of which were upregulated and 1514 of which were downregulated. Consequently, it can be inferred that old soybean leaves may possess a unique molecular regulatory mechanism to mitigate Mn toxicity stress.

Metal transporters have a pivotal part in alleviating the effectiveness of Mn toxicity in plantlet by means of facilitating the transport of Mn to other sites [17]. For example, PpNramp5 in peach (*Prunus persica*) is upregulated in response to Mn stress and has been shown to function as a Mn transporter when expressed heterologously in yeast (*Saccharomyces cerevisiae*) [57]. Rice OsNRAMP5 regulates Mn transport from the soil to internal root tissues and from the apoplast to the endodermis [58]. Knockout of *OsNRAMP5* leads to decreased rice yield and biomass, especially under low environmental Mn concentrations [59]. In this study, the expression of *natural resistance-associated macrophage protein* (*NRAMP*) (*Glyma.08G218200*), *ion transporter* (*ITP*) *Glyma.05G131000*), and *zinc transporter* (*ZIP*) (*Glyma.13G004400*) in old soybean leaves was observably upregulated responding to Mn poisoning, indicating its involvement in the response to Mn stress. Therefore, modulating the transcriptional levels of metallic ions transporters may represent an effective molecular mechanism for enhancing Mn tolerance in old soybean leaves under Mn toxicity stress.

Excessive Mn triggers oxidative stress through a series of processes, disrupting the homeostasis of plant tissue structure, with the activation of the antioxidant enzyme system being crucial for plant resistance to Mn stress [60,61]. The induction of antioxidase system gene expression and POD activity has been observed in cowpea (*Vigna sinensis*) and soybean responding to Mn toxicity, indicating that those responses possibly have a key part in regulating Mn stress [61,62]. The expression of *OsAPX8* in rice roots is enhanced by salt stress, while high salt concentrations decrease the expression of *OsAPX7*, indicating that APX may regulate plant abiotic stress tolerance [63]. In the present study, two *PODs* (*Glyma.09G277800* and *Glyma.18G211100*) were downregulated in old soybean leaves under high Mn stress. Hence, the downregulation of 2 *PODs* might contribute to enhancing the Mn toleration of old leaves in soybean.

Aux/IAA proteins, pivotal components of the auxin signal transduction pathway, play a significant role in the response to external environmental cues [64]. Mn stress has been shown to reduce the auxin content in *Arabidopsis* roots by downregulating the expression of *PIN7* and *PIN4*, inhibiting the auxin biosynthesis pathway [11]. Additionally, qRT-PCR results have demonstrated a significant decrease in the gene transcriptional levels connected with auxin synthesis under Mn toxicity stress [11]. In the present study, the expressions of two genes connected with auxin synthesis (*Glyma.17G258200*) and gibberellin regulatory protein (*Glyma.09G095200*) were significantly downregulated, indicating that Mn stress affects the expressing of genes relevant to auxin synthesis, leading to changes in the auxin content in old soybean leaves.

Many studies have begun to investigate the effects of glutathione S-transferase, chlorophyll enzyme, and lipoxygenase on plant development responding to metal ion poisoning [65,66,67]. In the present study, 16 genes involved in cellulose biosynthesis, 15 genes related to glutathione metabolism, 11 genes related to sugar transport, and 9 genes related to amino acid transport exhibited differential expression in old soybean leaves under Mn stress. These results suggest that these enzyme-related genes are involved in the response of old soybean leaves to Mn poisoning stress. However, the precise regulatory mechanisms of these enzyme-encoding genes responding to Mn poisoning stress have not yet been determined. Therefore, further exploration is needed to uncover the complex regulatory mechanisms involved in the responding of old soybean leaves to Mn stress.

In summary, the experimental findings demonstrated that an increase in the exogenous Mn concentration led to the inhibition of photosynthetic pigment synthesis in old soybean leaves and disrupted the homeostasis of internal tissue cells, significantly impacting the phenotype of these old leaves. Mn toxicity stress inflicted damage to cell membranes; heightened oxidative stress; and induced peroxidase activity, hormone synthesis, amino acid synthesis, cellulose synthesis, and ion transporter gene expression, thereby affecting tissue cell metabolism. However, these alterations in ion accumulation, physiological and biochemical parameters, and hormone biosynthesis had adverse effects on the normal growth of old soybean leaves.

**Figure 8:**
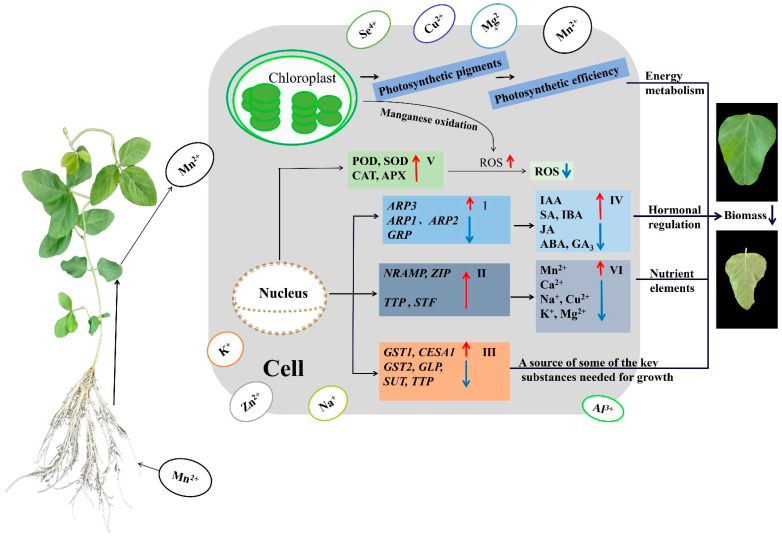
Regulation process of old soybean leaves coping with manganese toxicity stress. The red arrows indicate upregulated gene expression, enhanced substance content, or heightened activity of enzyme; blue arrows indicate downregulated gene expression or reduced substance content; Ⅰ: hormone-related genes; II: ion transporter-related genes; Ⅲ: glutathione, cellulose synthase, sugar, and amino acid transporters and other related genes; Ⅳ: hormone; V: antioxidant enzymes; VI: ion content.

Under high Mn toxicity, antioxidant enzymes in old soybean leaves were activated to control ROS levels within a certain range. However, the trend toward excessive ROS production could not be completely reversed, leading to irreversible damage to the tissue cells of old leaves. In conclusion, elevated Mn toxicity significantly compromised tissue cell integrity, nutrient uptake, hormone synthesis, and photosynthetic efficiency in old soybean leaves, resulting in hindered metabolism and growth and reduced aboveground biomass. The regulation procedure of Mn stress in old soybean leaves is depicted in Figure 8. These consequences highlight the presence of a more complex regulatory mechanism governing the responding of old soybean leaves to high Mn stress, necessitating further exploration.

## 4. Materials and Methods

### 4.1. Preparation of Plant Materials

The variety of soybean named Yuechun 03-3 (YC03-3) was developed by South China Agricultural University, and the plant experimental materials were cultivated at the Agricultural Research Institute of GDOU (Guangdong Ocean University) (east longitude: 110.300695, northern latitude: 21.151325). Soybean seeds with smooth surfaces, devoid of cracks, and uniform in size were carefully selected. These seeds were subjected to surface sterilization using a chlorine-based method involving 100 mL of 10% NaClO solution with the addition of 4.2 mL of 36% HCl, and this treatment was conducted over a duration of 14 h [68]. Following sterilization, the seeds were rigorously planted in quartz sand and nurtured for a period of 4 days.

Subsequently, soybean plants displaying intact roots, stems, and primary leaves with comparable growth potential were chosen and transplanted into 12-L plastic containers for hydroponic cultivation. An improved Hoagland nutrient solution was employed for hydroponics [39]. The nutrient mixture included the following components: 25 µM of MgCl_2_, 1500 µM of KNO_3_, 400 µM ofNH_4_NO_3_, 1200 µM of Ca(NO_3_)_2_·4H_2_O, 40 µM of Fe-EDTA(Na), 0.16 µM of (NH_4_)_5_MoO_24_·4H_2_O, 0.5 µM of CuSO_4_·5H_2_O, 500 µM of MgSO_4_·7H_2_O, 300 µM of (NH_4_)_2_SO_4_, 2.5 µM of NaB_4_O_7_·10H_2_O, 300 µM of K_2_SO_4_, 500 µM of KH_2_PO_4_, and 1.5 µM of ZnSO_4_·7H_2_O. All of the chemical reagents utilized were analytically pure (AR) in this study. Concurrently, varying concentrations of MnSO_4_ (5, 35, 100, 165, and 230 µM) from Solarbio (Solarbio, Beijing, China) were supplemented to the liquid nutrient solutions to induce Mn treatments. The normal group was exposed to a Mn concentration of 5 µM. Four independent experiments were conducted for each Mn concentration. The cultivation environment was maintained within the temperature maintaining at 25–28 °C, the pH value of the liquid nutrient solution was regulated to 5.0, the light cycle consisted of 12 h of light per day, and the light intensity was set at 2000 lux.

### 4.2. Determination of Photosynthetic Pigment Content

The assessment of photosynthetic pigment content in old leaves exposed to various Mn concentrations (5, 35, 100, 165, and 230 µM) was conducted following the methodology outlined by Porra et al. [69]. For each sample, 0.2 g of fresh leaf material was placed into a glass test tube. Subsequently, 10 mL of 95% anhydrous ethanol from Kermel, Tianjin, China, was added to the test tube. The glass test tube was then carefully wrapped in tinfoil and allowed to stand for 48 h. Following this period, the extracts were filtered, and the absorbency indexes at 663, 645, and 440 nm were tested using a double-beam ultraviolet–visible spectrophotometer (TU1901 from Pu Analysis, Beijing, China).

### 4.3. Measurement of Physiological Response Indices

The physiological indicators of the old leaves dealt with varying Mn concentrations (5, 35, 100, 165, and 230 µM) were assessed as follows: (1) Activity of SOD was determined using the NBT (nitrogen blue tetrazole) technique [70]. (2) Activity of POD was determined utilizing the (CH_3_O)C_6_H_4_OH (guaiacol) technique [71]. (3) APX activity was measured employing the sulfosalicylic acid method [72]. (4) CAT activity was assessed via spectrophotometry [73]. (5) Proline content was quantified using the sulfosalicylic acid method [72]. (6) MDA content was measured utilizing the thiobarbituric acid method [74].

### 4.4. Determination of Hormone Content

The methods for the determination of endogenous hormones and plant hormones extracted from soybean leaves subjected to varying concentrations of Mn (5 and 100 μM) after 15 days referred to the previous reports [75,76]. The concentrations of endogenous hormones, namely, IAA, IBA, GA3, JA, ABA, and SA, in mature soybean leaves were ascertained. The judgments of those phytohormones were implemented using HPLC (high-performance liquid chromatography) (AGILENT 1290, Santa Clara, CA, USA) in conjunction with MS/MS SCIEX-6500Qtrap (tandem mass spectrometry) (Allen Bradley, Milwaukee, WI, USA). The external reference materials employed, including IBA, SA, ABA, IAA, JA, and GA3, were all of chromatography grade purity (Sigma, St. Louis, MO, USA). The internal reference materials included D-IBA (deuterated IBA), D-SA (deuterated SA), D-ABA (deuterated ABA), D-JA (deuterated JA), D-GA3 (deuterated GA3), and D-IAA (deuterated IAA) (Sigma, St. Louis, MO, USA). The column was filled with C18 QuECherS (Ampere, Shanghai, China) material. The methyl alcohol and methyl cyanide utilized in the procedure were both of chromatographically pure grade (Merck KGaA, Darmstadt, Germany).

Preparation of the standard curve: First, 988 µL of methanol solution was supplemented in 1.5 mL centrifuge tubes, and 2 L of each 500 μg/mL of the hormone external standard stock solution was added, and then configured as the external standard master solution (1 µg/mL). Then, 990 µL of methyl alcohol solution was put in 1.5 mL centrifugal tubes, and 2 L of each 500 µg/mL of the hormone internal standard stock solution was added and vigorously whisked to make the internal standard master solution at a final concentration of 1 µg/mL. Finally, methanolic solutions were used to make standard curves with final concentrations of 0.1, 0.2, 0.5, 2, 5, 20, 50, and 200 ng/mL, with each concentration point containing 20 ng/mL of the hormone internal standard. According to the results of the HPLC–MS/MS measurements, the standard curve could be plotted, where the horizontal coordinate X was the concentration of the external standard divided by the corresponding internal standard, and the vertical coordinate Y was the peak area of the external standard divided by the corresponding internal standard.

Hormone extraction: Six kinds of hormones were extracted from old soybean leaves by crushing samples to be analyzed in liquid nitrogen and carefully weighing approximately 1 g of the samples in glass test tubes. Each hormone measurement was performed three times. A 10-fold amount of acetonitrile solution was supplemented, as well as 8 µL of the 20 ng/mL master solution of the matching internal standard. The old leaves samples were carefully extracted overnight at 4 °C and centrifuged for 5 min at a rotation speed of 12,000× *g* rpm. After the liquid supernatant was gained, the sediment was dealt with a fivefold amount of acetonitrile solution, extracted two times, and united with the supernatants. The sample received approximately 35 mg of C18 packing before being shocked violently for 30 s and centrifuged at a velocity of 10,000× *g* rpm for 5 min. The liquid supernatant was then eliminated. The samples were blown dry with nitrogen, dissolved respectively in 400 µL methyl alcohol, filtered through 0.22 µm needle filters (Millipore SLFGX13NL, Billerica, MA, USA), and reserved at −20 °C in a refrigerator (Ronshen BCD-218WD12NY, Foshan, China) for HPLC–MS/MS. The detailed gradient parameters for HPLC (Table S2), mass spectrum parameters (Table S3), and the selected monitoring indicators for protonation or deprotonation reactions of phytohormones (referred to Table S4) can be found in the Supplementary Materials.

### 4.5. Determination of the Ion Content

A total of 0.2 g of dried old soybean leaves subjected to different Mn concentrations (5 μM and 100 μM MnSO_4_) was individually weighed and placed into separate 50 mL glass boiling tubes (Dafeng, Shanghai, China). Subsequently, these boiling tubes were positioned within 50 mL polytetrafluoroethylene crucibles (Binzhenghong, Nanjing, China). Next, 5 mL of sulfuric acid (8 mM) was supplemented to each crucible (Hushi, Shanghai, China). These mixtures were left at room temperature for a 24 h period. The crucibles were subsequently transferred to a GS-type electric heating plate (Binzhenghong, Nanjing, China) and gradually heated to 180 °C for a 2.5 h digestion process. Subsequently, the samples were allowed to cool to room temperature, after which 2 mL of sulfuric acid (8 mM) and 2 mL of 30% H_2_O_2_ (Solarbio, Beijing, China) were supplemented. The crucibles were then maintained at 180 °C for 4 h and subsequently heated to 140 °C until all the liquid had completely evaporated. Finally, the remaining precipitated materials were fully dissolved in 1 mL of concentrated nitric acid, separately transferred to 25 mL colorimetric tubes (Xinpeng, Taizhou, China), and diluted with deionized water to the 25 mL mark. The concentrations of ions within the old soybean leaves were measured using ICP–AES PS7800 (inductively coupled plasma–atomic emission spectrometer) (Hitachi Limited, Tokyo, Japan) [77].

The standard curve was prepared using the National Non-Ferrous Metal and Electronic Materials Analysis and Testing Center’s (GNMM260194-2013) liquid standard sample with concentrations of 0, 0.5, 1, 5, 10, 20, 50, and 100 μg/L. The standard series solution was measured in accordance with the instrument’s parameters, and the standard curve was generated.

Finally, the element content of the sample was estimated using the following formula: W = ((X1 − X0) × V)/M (W: the element to be measured, the mass fraction was 10^−6^; X1: the concentration of the target compound in the sample digestion solution, in micrograms per liter; X0: the concentration of the target compound in the blank solution, in micrograms per liter; V: the volume of the sample digestion solution in liters; M: the sample weighing weight in grams).

### 4.6. Transcriptome Sequencing of Old Soybean Leaves

Old soybean leaves obtained from seedlings subjected to 15 days of treatment with both 5 µM Mn (normal concentration) and 100 µM Mn (toxic concentration) were utilized for extraction of RNA and construction of mRNA library. Transcription sequencing of the old soybean leaves was conducted following a formerly described technology [78]. Each sample test was carried out for three biological replicates. Extraction of RNA was performed using the Kit of MolPure Plant RNA (Yeasen, Shanghai, China). For the mRNA library construction, RNA samples were enriched with magnetic beads with oligo (dT). Subsequently, random primers were used for reverse transcription and fragmentation. After purification, the cDNA was treated with terminal repair, and PCR was utilized to amplify the complete library. The library was sequenced using Illumina platform, and sequencing strategy was PE150. To ensure the quality of the data, the original sequence (i.e., Raw Data) must be filtered to obtain a high-quality sequence (i.e., Clean Data). Then, sequence alignment was conducted using TopHat (version 2.0.12) to map the Clean Data to soybean reference genome (Williams 82.a2.v1) [79,80]. HTSeq (version 0.6.0) and fragments per kilobase of transcript per million reads mapped (FPKM) were adopted for the quantitation of gene expression levels, and the differentially expressed genes (DEGs) were identified by DESeq (version 1.16) and adjusted with q ≤ 0.05 and |log2 ratio| ≥ 1 [81]. These data were uploaded into Gene Expression Omnibus (https://www.ncbi.nlm.nih.gov/geo/) (accessed on 5 September 2023) under GSE118649. For accomplishGene Ontology (GO) analyses of functional enrichment, the DAVID (Database for Annotation, Visualization, and Integrated Discovery) (https://david.ncifcrf.gov/) (accessed on 7 September 2023) was adopted for implementation [82,83]. The summarized results of gene expression are presented in Table S4 and were derived from the transcriptome sequencing of old soybean leaves under Mn toxicity stress.

### 4.7. qRT-PCR Analysis

RNA extracted from old soybean leaves subjected to various Mn treatments was processed as described in Section 2.11. The RNA was isolated from the old leaves of soybean via the Kit of MolPure Plant RNA (Yeasen, Shanghai, China). DNaseI (Yeasen Biotechnology, Shanghai, China) was used to remove DNA contamination. Subsequently, cDNA was synthesized adopting the kit of Hifair^®^ II 1st Strand cDNA Synthesis (Yeasen Biotechnology, Shanghai, China). Quantitative RT-PCR (qRT-PCR) was carried out with the help of a fluorescent quantitative PCR instrument CFX Connect Optics Module (Bio-Rad Company, Hercules, CA, USA) [38]. The 20 μL qRT-PCR mixture consisted of 8 μL of ddH2O, 10 μL of Hieff UNICON Universal Blue qPCR SYBR Green Master Mix (Yeasen Biotechnology, Shanghai, China), 0.5 μL of forward primer, 0.5 μL of reverse primer, and 1 μL of cDNA. The qRT-PCR procedure involved maintaining at 95 °C for 30 s; keeping at 95 °C for 5 s; maintaining at 58 °C for 60 s; and a final maintaining at 72 °C for 30 s. The *GmEF* (*Glyma.17G186600*) was selected as the internal reference gene. The relative transcription level was determined based on the ratio of target gene to reference gene, as formerly report described in the Results and Analysis section [84]. The primers applied to qRT-PCR are displayed in Table S5.

### 4.8. Analysis of Data

Data analysis was implemented by application of Microsoft Excel 2010 (Microsoft, Washington, DC, USA) and SPSS Statistics 26 (SPSS, Chicago, IL, USA). Significant differences in data were judged through the use of the *t*-test and Duncan multiple comparison method [39,84].

## 5. Conclusions

This study comprehensively examined the effect of Mn toxicity on old soybean leaves through a combination of physiological and molecular approaches. Mn toxicity hindered the biosynthesis of photosynthetic pigments in old soybean leaves, resulting in decreased photosynthetic efficiency and ultimately leading to a reduction in the biomass and overall plant growth of old soybean leaves. Additionally, the excessive accumulation of Mn in old leaves triggered the activation of the antioxidant enzyme system, disrupted the uptake of various metal ions, and interfered with hormone synthesis, thereby disturbing the internal equilibrium within old soybean leaves. Furthermore, transcriptome sequencing analysis revealed 2258 genes in old soybean leaves that responded to Mn toxicity, 744 of which were upregulated and 1514 of which were downregulated. To validate these results, qRT-PCR was performed on fifteen selected genes, including those encoding *hormones*, *glutathione metabolism*, *cellulose biosynthesis*, *amino acid transport*, and *glycometabolism*, yielding findings consistent with the transcriptome data. The consequences of this research contribute valuable suggestion into the mechanisms underlying the responding of old soybean leaves to Mn toxicity, offering a foundation for further investigations into the specific molecular regulatory pathways involved in the response of old soybean leaves to Mn poisoning.

## Figures and Tables

**Table 1:** Identification of genes related to hormone synthesis.

Gene ID	log2FoldChange	Description
*Glyma.01G137500*	2.12	Auxin-responsive protein
*Glyma.02G125600*	−2.77	GH3 auxin-responsive promoter
*Glyma.03G029600*	1.66	Auxin-responsive protein
*Glyma.06G158700*	1.43	Auxin-responsive protein
*Glyma.07G043000*	1.36	Dormancy/auxin-associated protein
*Glyma.07G051700*	1.18	Auxin-responsive protein
*Glyma.10G151000*	2.27	Dormancy/auxin-associated protein
*Glyma.12G035800*	2.12	Auxin-responsive protein
*Glyma.12G071000*	−1.30	Auxin-responsive protein
*Glyma.13G150100*	−2.44	Auxin-responsive protein
*Glyma.15G076500*	2.23	Dormancy/auxin-associated protein
*Glyma.17G046200*	−4.17	Auxin-responsive protein
*Glyma.17G165300*	−2.72	GH3 auxin-responsive promoter
*Glyma.02G007300*	−3.34	AUX/IAA family
*Glyma.10G180000*	1.14	AUX/IAA family
*Glyma.13G354100*	−1.40	AUX/IAA family
*Glyma.15G012700*	−1.45	AUX/IAA family
*Glyma.02G142500*	−1.37	AUX/IAA family
*Glyma.03G158700*	−1.31	AUX/IAA family
*Glyma.04G046900*	−1.65	AUX/IAA family
*Glyma.19G161000*	−1.30	AUX/IAA family
*Glyma.06G185300*	−3.81	Gibberellin-regulated protein
*Glyma.09G095200*	−1.37	Gibberellin-regulated protein
*Glyma.14G087200*	−1.73	Gibberellin-regulated protein
*Glyma.17G258200*	−2.25	Gibberellin-regulated protein
*Glyma.02G297700*	−3.09	GRAS domain family
*Glyma.06G265500*	−2.95	GRAS domain family
*Glyma.08G325900*	−2.13	GRAS domain family
*Glyma.12G018100*	1.64	GRAS domain family
*Glyma.12G137700*	−3.11	GRAS domain family
*Glyma.12G216100*	−1.96	GRAS domain family
*Glyma.15G037200*	1.26	GRAS domain family
*Glyma.17G007600*	−3.53	GRAS domain family
*Glyma.17G160800*	1.47	GRAS domain family
*Glyma.18G081100*	−1.98	GRAS domain family

**Table 2:** Identification of glutathione metabolism genes.

Gene ID	log2FoldChange	Description
*Glyma.01G106100*	−1.05	Glutathione S-transferase
*Glyma.02G024600*	3.98	Glutathione S-transferase
*Glyma.02G024800*	4.94	Glutathione S-transferase
*Glyma.04G107500*	−1.12	Glutathione S-transferase
*Glyma.06G193500*	1.70	Glutathione S-transferase
*Glyma.07G139700*	−1.39	Glutathione S-transferase
*Glyma.07G139800*	−2.87	Glutathione S-transferase
*Glyma.07G140400*	−1.14	Glutathione S-transferase
*Glyma.15G251500*	−1.82	Glutathione S-transferase
*Glyma.15G251600*	−1.26	Glutathione S-transferase
*Glyma.15G251700*	−3.19	Glutathione S-transferase
*Glyma.15G251900*	1.21	Glutathione S-transferase
*Glyma.18G190200*	−1.78	Glutathione S-transferase
*Glyma.05G207100*	−1.24	Glutathione peroxidase
*Glyma.11G024000*	−1.02	Glutathione peroxidase

**Table 3:** Identification of cellulose synthase genes.

Gene ID	log2FoldChange	Description
*Glyma.04G063800*	−1.26	Cellulose synthase
*Glyma.06G065000*	−1.67	Cellulose synthase
*Glyma.06G225400*	−5.80	Cellulose synthase
*Glyma.06G307900*	4.50	Cellulose synthase
*Glyma.08G088400*	−4.22	Cellulose synthase
*Glyma.08G117500*	−3.70	Cellulose synthase
*Glyma.09G051100*	−1.83	Cellulose synthase
*Glyma.12G191700*	−2.85	Cellulose synthase
*Glyma.13G310300*	1.08	Cellulose synthase
*Glyma.15G157100*	−1.88	Cellulose synthase
*Glyma.01G232500*	−2.62	Cellulose synthase
*Glyma.11G010400*	−1.57	Cellulose synthase
*Glyma.02G205800*	−2.83	Cellulose synthase
*Glyma.04G255400*	1.08	Cellulose synthase
*Glyma.06G225500*	−1.89	Cellulose synthase
*Glyma.06G324300*	1.10	Cellulose synthase

**Table 4:** Identification of amino acid transport-related genes.

Gene ID	log2FoldChange	Description
*Glyma.01G084800*	−1.41	Transmembrane amino acid transporter protein
*Glyma.02G260100*	−1.02	Transmembrane amino acid transporter protein
*Glyma.05G043100*	1.20	Transmembrane amino acid transporter protein
*Glyma.09G238100*	−2.72	Transmembrane amino acid transporter protein
*Glyma.10G201600*	−1.81	Transmembrane amino acid transporter protein
*Glyma.11G097000*	−2.11	Transmembrane amino acid transporter protein
*Glyma.11G226000*	−3.11	Transmembrane amino acid transporter protein
*Glyma.20G188800*	−2.74	Transmembrane amino acid transporter protein
*Glyma.14G010300*	1.70	Transmembrane amino acid transporter protein

**Table 5:** Identification of glycometabolism genes.

Gene ID	log2FoldChange	Description
*Glyma.02G075000*	−1.57	Sugar (and other) transporter
*Glyma.07G086000*	−2.86	Sugar (and other) transporter
*Glyma.07G189500*	−2.13	Sugar (and other) transporter
*Glyma.08G009900*	−1.85	Sugar efflux transporter for intercellular exchange
*Glyma.11G004800*	−1.24	Sugar (and other) transporter
*Glyma.15G071800*	−1.35	Sugar (and other) transporter
*Glyma.16G138800*	−1.00	Sugar (and other) transporter
*Glyma.16G156800*	−1.50	Sugar (and other) transporter
*Glyma.08G135800*	−1.92	Nucleotide-sugar transporter
*Glyma.11G119500*	−1.45	Sugar (and other) transporter
*Glyma.13G284900*	−1.50	Sugar (and other) transporter

## Data Availability

Data are contained within the article and Supplementary Materials.

## Supplementary Materials

The following supporting information can be downloaded at: https://www.mdpi.com/article/10.3390/ijms25105341/s1.
